# Purification and Characterization of Haloalkaline, Organic Solvent Stable Xylanase from Newly Isolated Halophilic Bacterium-OKH

**DOI:** 10.1155/2014/198251

**Published:** 2014-09-08

**Authors:** Gaurav Sanghvi, Mehul Jivrajani, Nirav Patel, Heta Jivrajani, Govinal Badiger Bhaskara, Shivani Patel

**Affiliations:** ^1^Department of Pharmaceutical Sciences, Saurashtra University, Rajkot, Gujarat 360 005, India; ^2^Department of Biotechnology, M. & N. Virani Science College, Rajkot, Gujarat 360 005, India; ^3^Department of Pharmacology and Toxicology, B. V. Patel Pharmaceutical Education and Research Development (PERD) Centre, Sarkhej-Gandhinagar Highway, Thaltej, Ahmedabad, Gujarat 380 054, India; ^4^Department of Biochemistry and Molecular Biology, Miller School of Medicine, University of Miami, Miami, FL 33136, USA; ^5^Department of Biochemistry, Saurashtra University, Rajkot, Gujarat 360 005, India

## Abstract

A novel, alkali-tolerant halophilic bacterium-OKH with an ability to produce extracellular halophilic, alkali-tolerant, organic solvent stable, and moderately thermostable xylanase was isolated from salt salterns of Mithapur region, Gujarat, India. Identification of the bacterium was done based upon biochemical tests and 16S rRNA sequence. Maximum xylanase production was achieved at pH 9.0 and 37°C temperature in the medium containing 15% NaCl and 1% (w/v) corn cobs. Sugarcane bagasse and wheat straw also induce xylanase production when used as carbon source. The enzyme was active over a range of 0–25% sodium chloride examined in culture broth. The optimum xylanase activity was observed at 5% sodium chloride. Xylanase was purified with 25.81%-fold purification and 17.1% yield. Kinetic properties such as *Km* and *V*max were 4.2 mg/mL and 0.31 *μ*mol/min/mL, respectively. The enzyme was stable at pH 6.0 and 50°C with 60% activity after 8 hours of incubation. Enzyme activity was enhanced by Ca^2+^, Mn^2+^, and Mg^2+^ but strongly inhibited by heavy metals such as Hg^2+^, Fe^3+^, Ni^2+^, and Zn^2+^. Xylanase was found to be stable in organic solvents like glutaraldehyde and isopropanol. The purified enzyme hydrolysed lignocellulosic substrates. Xylanase, purified from the halophilic bacterium-OKH, has potential biotechnological applications.

## 1. Introduction 

Biomass has been recognized as one of the major world renewable energy sources in which cellulose and hemicellulose are considered as its major fraction [1]. Hemicellulose represents a group of plant polysaccharides with different structures and different monosaccharide composition, which can be present in various amounts or traces depending on the natural source [2]. Xylan is the principal hemicelluloses and major plant cell wall polysaccharide component, composed mainly of D-xylose. It is a heteropolysaccharide with a homopolymeric chain of 1,4,*β*-d-xylosidic linkages with the backbone comprising of O-acetyl, α-L-arabinofuranosyl, and 1, 2-linked glucuronic or 4-O-methylglucuronic acid [3]. Xylanases (EC 3.2.1.8) randomly hydrolyze the *β*-1,4-glycosidic bonds of xylan to produce xylooligomers of different lengths [4]. Many kinds of xylanases have been isolated from various microorganisms like fungi, bacteria, actinomycetes, and yeasts [5].

In the recent years, microorganisms from extreme conditions have been the focus of researchers attention as enzymes from extremophilic microorganisms can withstand harsh conditions like extreme temperature, salt, alkaline condition, and so forth. Extremophiles can be classified into thermophiles, psychrophiles, acidophiles, alkaliphiles, halophiles, and others [6]. Halophiles have gained attention due to their extensive mechanism of adaptation to extreme hypersaline environments and are differentiated based on salinity into nonhalophile (<1.2% NaCl), slight halophile (1.2–3%), moderate halophile (3–15%), and extreme halophile (>15% NaCl) [7]. Halophiles are the most likely source of such enzymes, because not only their enzymes are salt-tolerant, but many are also thermotolerant [8]. Furthermore, exoenzymes from halophiles are not only interesting from the basic scientific viewpoint but they may also be of potential interest in many industrial applications, owing to their stability and activity at low water activities [9, 10].

Currently, major application of xylanase is in pulp and paper industries where xylanases replace chemical bleaching agents, which results in greater brightness in pulp. Most industrial pulping is done at high temperature and under alkaline conditions, hence requiring xylanases to be operationally stable under such conditions. To meet the specific industry's needs, an ideal xylanase should equipped with specific properties, such as good pH and thermal stability, high specific activity, and strong resistance to metal cations and chemicals, are also pivotal factors to the applications. However, the great majority of xylanases reported so far are neither active nor stable at both high temperature and high pH [11]. Thus, much research interest has been generated in the production of xylanase under halophilic conditions [12].

In the present study, production and characterization of haloalkaline thermostable xylanase by a newly isolated, halophilic bacterium-OKH is reported.

## 2. Material and Methods

### 2.1. Isolation and Maintenance of Microorganism

The halophilic bacterium-OKH was isolated from sediments collected from salt salterns around Mithapur. Culture was grown on agar plates containing 0.5% (w/v) Birchwood xylan, 0.5% yeast extract in mineral salt medium containing (g/L) NaCl 150, MgCl_2_ 5.0, K_2_SO_4_ 0.2, and agar with pH adjusted to 9.0. After 96 hrs, plates were flooded with 0.1% Congo red solution for 15–20 mins and then destained with 1 M NaCl for 10–15 mins [13]. The colonies showing clear zone of hydrolysis were picked and used for xylanase production. Based on the zone of clearance, xylanase from halophilic bacterium-OKH was selected for further studies.

### 2.2. Bacterial Identification and Phylogenetic Analysis

The morphological, cultural, and biochemical characteristic of the isolated strain was studied according to Bergey's Manual of Determinative Bacteriology [14]. Genomic DNA of halophilic bacterium-OKH was isolated by SDS lysozyme method [15] with slight modification in method by adding extra P:C:I and C:I step to remove high amount of protein impurities obtained. PCR amplification of 16srRNA was performed using the forward 5′-AGAGTTTGATCCTGGCTCAG-3′ and reverse primer 5′-CAACCTTGTTACGACT-3′, respectively. The obtained PCR product was sequenced and 16srRNA gene sequence was compared with GenBank submissions using BLASTn programme. The phylogenetic analysis was done by RDP PHYLIP software.

### 2.3. Enzyme Production

2 mL of 96 hr old culture was inoculated to 250 mL Erlenmeyer flasks containing the following media (g/L): yeast extract 3.0, NaCl 150, MgCl_2_ 5.0, K_2_SO_4_ 0.2, and CaCl_2_ 0.02 gm, respectively. Media were supplemented by 0.5 gm of Birchwood xylan and 1 gm of corn cobs as substrates. Production media were autoclaved at 121°C for 15 mins at 15 lbs pressure. Flasks were incubated in rotary shaker at 120 rpm. After every 24 hrs of interval, flasks were removed and the content was centrifuged at 10,000 rpm for 20 mins. The crude supernatant was used for xylanase assay.

### 2.4. Study of Physicochemical Factors on Xylanase Production

#### 2.4.1. Effect of Carbon and Nitrogen Sources on Enzyme Production

Carbon sources such as glucose, maltose, lactose, arabinose, glucose, galactose, sucrose, fructose, mannose, and xylose were used in 1% (w/v) to check the effect of these supplements on enzyme production. Additionally, various concentrations of rice straw, wheat straw, sugarcane bagasse, corn cobs, rice husks, groundnut shells, and saw dust were also used to enhance the production of xylanase. In case of nitrogen sources, effect of both organic and inorganic nitrogen sources on enzyme production was studied. Peptone, malt extract, beef extract, and yeast extract were used as organic nitrogen sources, whereas for inorganic nitrogen sources, urea, ammonium sulphate, and sodium nitrate were used.

#### 2.4.2. Effect of NaCl, pH, and Temperature on Xylanase Production

To study the effect of NaCl on enzyme production, organism was cultivated at different NaCl concentrations ranging from 0 to 25%. Effect of pH and temperature on enzyme production was evaluated by varying pH (2.0–10.0) and temperature (10–70°C) of the production medium. Extracellular xylanase activity was measured in culture supernatant.

### 2.5. Xylanase Assay

Xylanase activity was determined at 37°C for 30 min in 0.05 M Tris-HCl buffer (pH 9.0) by DNSA (3,5-dinitrosalicylic acid) [16]. In blank, enzyme was added after adding DNSA reagent. The absorbance was measured at 540 nm. One unit of xylanase activity was defined as the amount of enzyme produced 1 *μ*mol of xylose equivalent per minute under specified conditions. Protein concentration was estimated by Lowry's method [17] using BSA (bovine serum albumin) as the standard.

### 2.6. Purification of Xylanase

All the purification steps were carried out at 4°C unless stated otherwise. The crude enzyme was subjected to 0–80% ammonium sulphate precipitation. The precipitated protein was collected by centrifugation (10,000 rpm) and dissolved in 0.05 M Tris-HCl buffer (pH 9.0). Collected fraction was dialysed and concentrated using rotary vacuum evaporator. Dialyzed sample was loaded on DEAE cellulose column (10 cm × 10 cm) and fractions were eluted at flow rate of 10 mL/hr. Fractions were eluted by linear gradient of 0-1 M NaCl. Fraction with maximum activity in ion exchange chromatography was further purified by size exclusion chromatography. Preequilibrated column of Sephadex G-100 was used for size exclusion chromatography. 1 mL fraction was collected at a flow rate of 10 mL/hr. Protein concentration of each fraction was determined by measuring OD at 280 nm. The purified fractions were checked for purity on SDS PAGE.

### 2.7. SDS PAGE and Zymogram Analysis

Homogeneity and molecular weight of the purified xylanase were determined by using 12% SDS PAGE as described by Laemmli [18]. Protein bands were visualised by staining with silver stain. The molecular weight standard used was the medium molecular weight marker for SDS electrophoresis procured from Genei (India). Zymogram analysis for xylanase was carried out as described by Hung et al. [19].

### 2.8. Influence of pH, Temperature, and Salinity on Xylanase Activity and Stability

The optimal temperature of the purified xylanase was determined in 0.05 M Tris-HCl buffer (pH 9.0) at a temperature range of 10–70°C. To evaluate stability, the enzyme solution was incubated at temperature the range of 10–70°C for 24 hours. Percentage relative enzyme activity was recorded at 4-hour intervals during 24-hour incubation.

The optimal pH of the purified xylanase was determined by measuring the activity between the pH 3.0 and 11.0. Three buffers (0.05 M) were utilized. Sodium acetate buffer was used for pH 3–5, sodium phosphate buffer for pH 4–7, and Tris-HCl buffer for pH 8–11. To test stability of purified xylanase, enzyme solution was incubated in 0.05 M Tris-HCl buffer (pH 9.0) for 24 hours. Aliquots were withdrawn at an interval of 4 hours. The xylanase activity was measured according to the standard assay method.

The optimal salt concentration for purified xylanase was determined in 0.05 M Tris-HCl buffer (pH 9.0) containing various concentrations of NaCl (0–30% w/v) concentrations. For stability, purified xylanase was incubated with 0.05 M Tris buffer (pH 9.0) with salinity in the range of 0–30% for 24 hours at 37°C. Each assay was presented as the average of three trials.

### 2.9. Effect of Metal Ions and Organic Solvents on Xylanase Activity

Effect of various metal ions such as HgCl_2_, MnCl_2_, CuCl_2_, CoCl_2_, AgNO_3_, ZnCl_2_, FeCl_2_, NiCl_2_, and NH_4_Cl was studied by adding each metal ion at two different concentrations (2 mM and 5 mM) in reaction mixture. Thereafter, the residual enzyme activities were determined under the standard assay conditions. Activity in the absence of metal ions was considered as 100%. To evaluate enzyme stability in organic solvent, different organic solvents like methanol, acetone, acetic anhydride, isopropanol, and glutaraldehyde were used. The enzyme activity was determined under standard assay conditions.

### 2.10. Storage Stability

To determine storage stability, enzyme was kept under different conditions with different time intervals; that is, it has been kept at room temperature for 3-4 days; it has been kept at storage temperature for one month and enzyme activity was checked. The kinetic constants,* Km* and* V*max, were estimated using linear regression plots of Lineweaver and Burk [20].

### 2.11. Application of Xylanase

Various lignocellulosics substrates like wheat straw, rice straw, and the commercial paper pulp samples sugarcane bagasse were saccharified by crude xylanase [21]. Each substrate (100 g/L of 0.05 M Tris-Cl, pH 9.0) was mixed with 50 mL of crude enzyme preparation. Saccharification was performed in shake flasks (120 rpm) at 37°C for 24 and 48 hours. The supernatants were assayed for estimation of reducing sugar.

### 2.12. Statistical Analysis

All the data were represented as average of least three independent experiments. Data have been represented as mean ± standard deviation.

The GenBank accession number of the sequence reported in this paper is EF063150.

## 3. Results and Discussion

### 3.1. Characterisation of Bacterial Strain

Halophilic bacteria are metabolically more versatile than the Archaea and their enzymatic activities are more diverse. To suit the industrial requirement halophilic bacteria are perfect resource to be used as it produces salt tolerant enzymes which are resistant to low pH. The halophilic bacterium-OKH used in the present study was isolated from soil sample collected near Mithapur, Gujarat, India. It is Gram-positive, rod shaped, translucent, and nonmotile bacteria which is catalase positive and hydrolysed gelatin and casein. It is sensitive to teicoplanin and chloramphenicol antibiotic. However, it is resistant to bacitracin, metronidazole, cefpodoxime, levofloxacin, tetracycline, and streptomycin (Table 1). It was able to ferment sugars like glucose, sucrose, xylose, and lactose without gas production.

**Figure 1:**
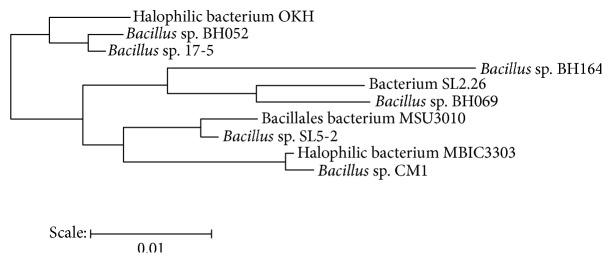
Dendrogram showing phylogenetic position of halophilic bacterium-OKH.

The result of the RDP Seqmatch and BLAST clearly showed that 16s rRNA gene sequence of isolate was distinct from the data available in the database. The 16s rRNA sequence showed ~90% identity to the* Bacillus *sp. BHO502. Henceforth, isolate belongs to class unclassified* Bacillus *sp. and designated as halophilic bacterium-OKH in current study as sample was collected from Okha region (near Mithapur) in Gujarat (Figure 1).

### 3.2. Growth Characteristics and Xylanase Production from Halophilic Bacterium-OKH

Most of the halophiles are slow growing. Production of xylanase started in early log phase and increased till late stationary phase but after that it declined. These results indicate that xylanase production was independent of growth phase which is in harmony with earlier reports on xylanase production by* Chromohalobacter* sp. [22].

#### 3.2.1. Effect of NaCl, pH, and Temperature on Enzyme Production

**Figure 2:**
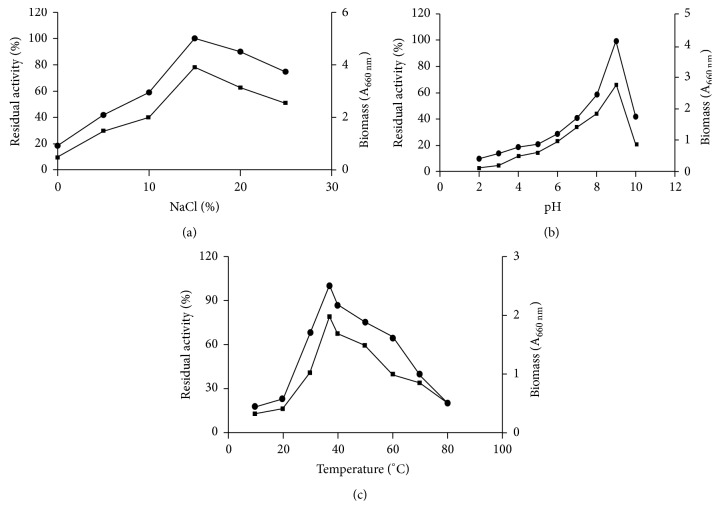
Effect of NaCl (a), pH (b), and temperature (c) on the growth and xylanase production by halophilic bacterium-OKH. Growth is represented by squares whereas xylanase activity is represented by circles.

Facilitated growth of OKH was observed in wide range of salinity from 5 to 20%. Highest growth and xylanase activity were obtained at pH 9.0 after 72 hours of incubation at 15% NaCl. The strain-OKH was found to be moderately halophilic in nature as no growth was observed in absence of NaCl. There was marked to be increased in activity with an increase in concentration of NaCl up to 15% and a further increase in NaCl concentration decline growth as well as production of xylanase (Figures 2(a) and 2(b)). This observation is in agreement with other halophilic organisms, namely,* Gracilibacillus *sp. TSCPVG[12] and* Chromohalobacter *sp.TPSV 101[22], where increase in salinity above optimum decreases enzyme production.

Since enzymes are very sensitive to pH, determination of the optimal pH is essential for xylanase production. In the present study, the effect of pH on production of enzyme was thus studied by carrying out fermentation over a wide range of pH (2.0–10.0). The production of xylanase was found to be highest at pH 9.0. There are reports of maximum xylanase production by halophiles from pH 7.5 to 9.0. It is evident from the data that xylanase from OKH is alkali-tolerant and offers use in pulp and paper industries. Maximal activity (28.14 U/mL) was observed at a temperature of 37°C. The optimal temperature for xylanase production by various halophiles has been previously studied; they have a wide range of temperature preferences depending upon nature of adaptation and salt requirements [23]. In the present study, OKH, being a mesophilic species, showed an optimal temperature at 37°C for maximal enzyme production (Figure 2(c)).

#### 3.2.2. Effect of Carbon and Nitrogen Sources on Enzyme Production

In the present study, oat spelt xylan was proven to be the best carbon sources for xylanase production followed by Birchwood xylan. Among all other carbon sources, supplementation of xylose increases the yield of xylanase whereas sucrose and lactose did not support growth as well as xylanase production. Increased yield of xylanase production by xylose supplementation was reported in the strain* Bacillus pumilus* GESF-1 [24]. To attain cost-effective production, different agro residues were used as substrate for xylanase production. The effective utilization of such agricultural wastes not only solves environmental problems but also promotes the economic value of the agricultural products. Appreciable xylanase activity was observed using 2% corn cobs as a carbon source (Table 2). Increased level of xylanase production using corn cobs may be due to its low lignin content and higher sugar content as compared to other substrates. Sugarcane bagasse and wheat straw also increase xylanase production. Similar reports have shown xylanase induction using lignocellulosic substrate in strains of* Cellulomonas flavigena* [25],* Staphylococcus *sp. [26], and* Bacillus pumilus *GESFI [24].

**Figure 3:**
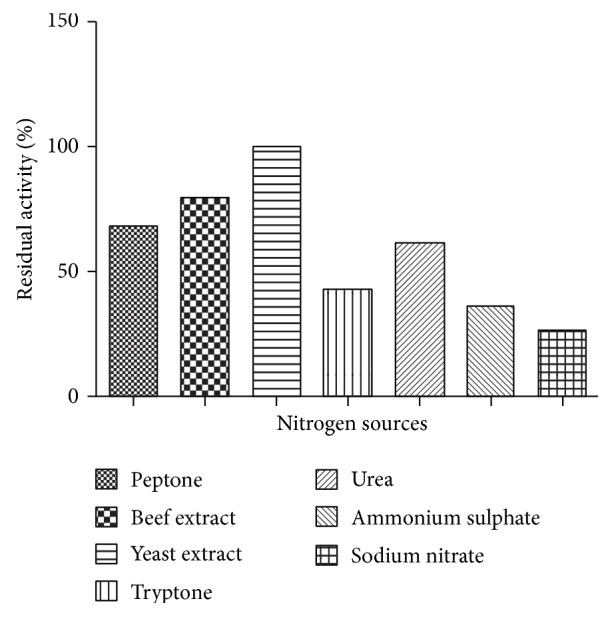
Effect of nitrogen sources on enzyme production.

Xylanase with minimal cellulases can be produced using low nitrogen to carbon ratio. Therefore, effect of concentration of nitrogen on production of enzyme is very important. Effect of nitrogen source on xylanase production is shown in Figure 3 (Figure 3). Very less activity was observed on supplementation of inorganic nitrogen sources compared to other organic nitrogen sources. Xylanase production was also supported by urea. Among organic nitrogen sources, yeast extract, peptone, tryptone, and beef extract resulted in better growth and xylanase production. Similar behavior has been reported in* Chromohalobacter *sp. TPSV 101 where organic nitrogen sources gave maximum xylanase production [12].

### 3.3. Purification of Xylanase

**Figure 4:**
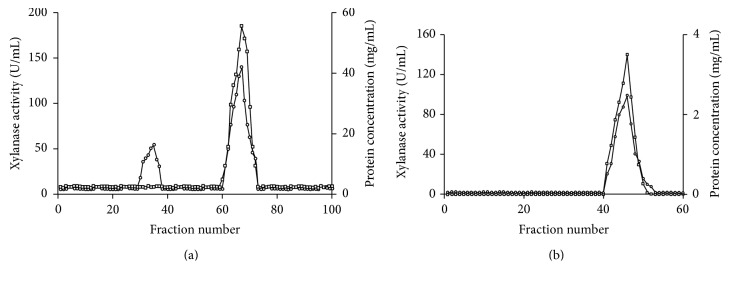
(a) Ion exchange chromatography. (b) Sephadex G-100 gel filtration chromatography of pooled, active fraction from ion exchange chromatography. Protein concentration is represented by circles whereas xylanase activity is represented by square.

**Figure 5:**
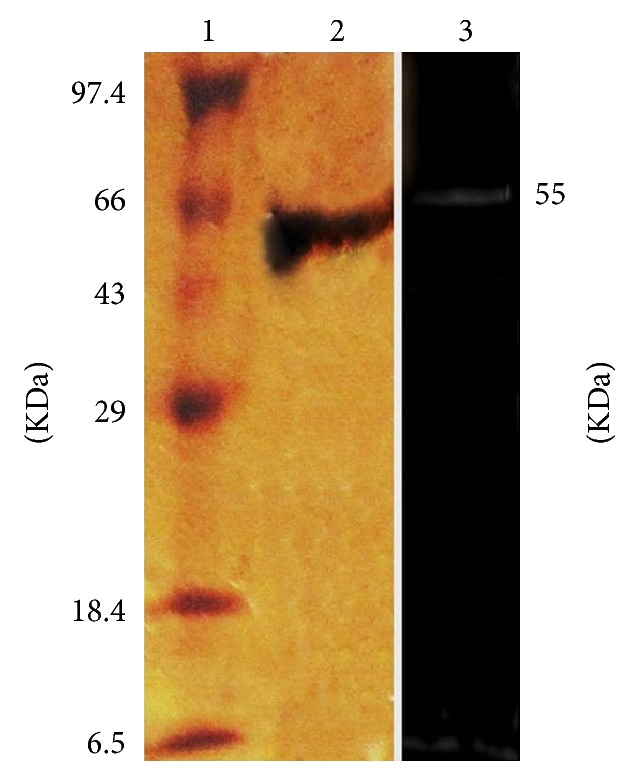
SDS-PAGE and zymogram analysis of purified xylanase from halophilic bacterium-OKH. Lane 1: marker, lane 2: xylanase in 12% SDS-PAGE, and lane 3: zymogram analysis of xylanase activity.

The crude enzyme was precipitated using ammonium sulphate to 80% saturation. The protein was purified by ion exchange DEAE Cellulose column and Sephadex G-100 gel filtration chromatography (Figure 4). The active fractions of purified ion exchange column were injected into Sephadex G-100 column. The purification has been summarized in Table 3 (Table 3). The purified enzyme xylanase exhibited 28.14 U/mg specific activities. Overall recovery of 17.1%- and 25.8-fold purity was observed. In case of* Gracilibacillus strain* acetone precipitated xylanase showed specific activity of 46.1 U/mg with 7% yield. Similar findings were reported in* Bacillus pumilus* sp. where 21-fold purity was observed with 2% yield. The purified enzyme showed a single band on SDS PAGE with a molecular mass of 55 KDa (Figure 5). The zymogram of xylanase exhibited a significant activity band that corresponds to result of SDS PAGE. High molecular weight xylanase (62 KDa) has been reported in strain Cl8 [23].

### 3.4. Effect of NaCl, pH, and Temperature on Enzyme Activity and Stability

**Figure 6:**
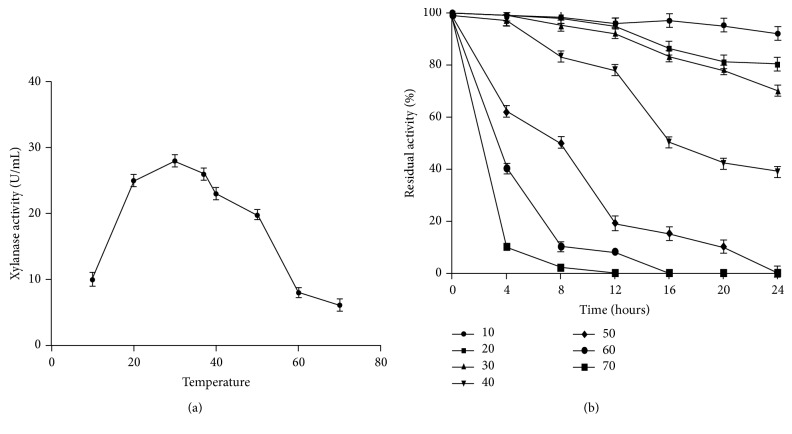
Graph showing (a) effect of temperature on enzyme activity. (b) Thermal stability of xylanase activity of halophilic bacterium-OKH. The values represent averages from triplicate experiments.

The results in Figure 6(a) demonstrate that the optimal temperature of purified xylanase was 37°C and it was stable in temperature range of 10–50°C. The enzyme activity declined rapidly as the temperature increased above 50°C and 15% of the activity was retained at 60°C after 4 hours of incubation (Figure 6(b)). In comparison,* Bacillus pumilus* GESF1 xylanase showed maximum activity at 40°C and retained about 80% at 60°C [24]. The xylanase of* Gracilibacillus *sp. TSCPVG, a moderate halophile, had the highest activity retained at 60°C whereas 83% of activity retained at 55°C and 61% of activity retained at 50°C, respectively [12]. Two extremely halophilic strains SX15 and CL8 also showed maximum activity at 60°C and 30°C [23, 27].

**Figure 7:**
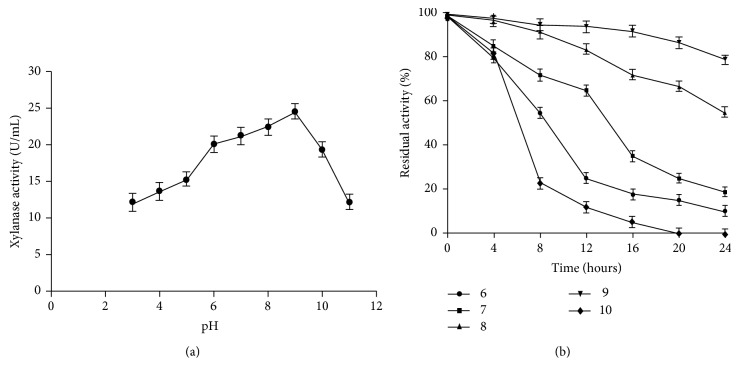
Graph showing (a) effect of pH on the activity of xylanase of halophilic bacterium-OKH. (b) pH stability of xylanase activity of halophilic bacterium-OKH. The values shown represent averages from triplicate experiments.

**Figure 8:**
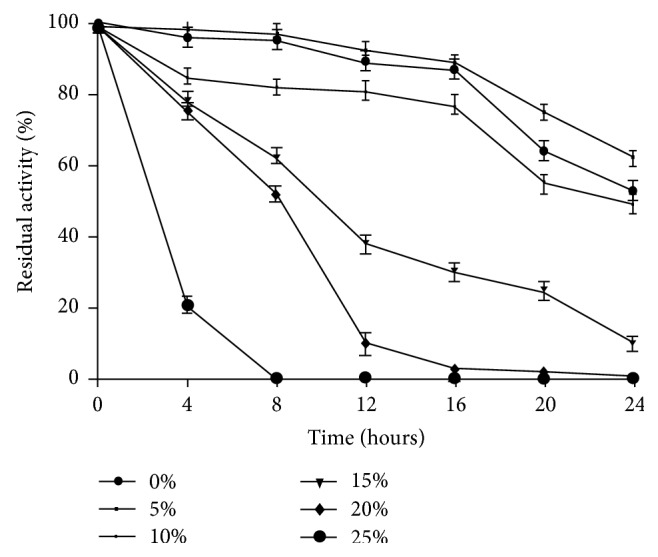
Graph showing NaCl stability of xylanase residual activity of halophilic bacterium-OKH at various time intervals.

Strain OKH xylanase exhibited maximal activity at pH 9.0 (Figure 7(a)). It was stable over a wide range of pH ranges from pH 6.0 to 10.0. About 35% of activity was observed at pH 6.0 after incubation of 8 hours. However, xylanase retained 80% of activity at pH 10.0 after 12 hours of incubation (Figure 7(b)).* Bacillus* sp. NG 27 showed maximum xylanase activity at pH 8.4 [28]. Similar to our finding, strain* Bacillus halodurans* showed maximum activity at pH 9.0 [11]. Xylanase was active over a broad range of NaCl concentration of 0–25% with optimal concentration (5%). At NaCl concentration of 25%, the enzyme retained 22% of its activity (Figure 8). Similar description has also been reported from* Bacillus pumilus* [24].

### 3.5. Effect of Additives on Enzyme Activity

Effect of metal ions and effectors are summarized in Table 4 (Table 4). Xylanase was not affected by addition of metal ions such as Ca^2+^, Mg^2+^, and Mn^2+^, but it was inhibited by other metal ions such as Ag^2+^, Hg^2+^, Fe^3+^, Ni^2+^, and Zn^2+^. Similar findings on inhibitory effect of metal ions on xylanase activity have also been reported from halophilic bacterium CL8 strain [23]. On the contrary, Zn^2+^ has stimulatory effect on xylanase from TSPVS strain while Mn^2+^ has been reported to inhibit xylanase activity of* Bacillus* sp. K-1 and* Bacillus halodurans* S7, respectively [11, 29].

Among the effectors tested, *β*-mercaptoethanol increased the activity considerably, indicating that reduced cystine residues are not involved, similar to xylanase of* Bacillus *sp. SPS-0 [30]. EDTA inhibited the activity suggesting that xylanase was metal ion dependent. Similar finding was reported in* Bacillus pumilus* sp. [24].

### 3.6. Effect of Organic Solvents on Xylanase Activity

To date, the use of halophilic extremozymes in organic solvents has been limited to very few enzymes [31]. The influences of different organic solvents on xylanase activity are shown in Table 5 (Table 5). Organic solvents like methanol, acetone, acetic anhydride, isopropanol, and glutaraldehyde were used to evaluate xylanase activity. Significant decrease in enzyme activity was found in the presence of 10% (v/v) solvents. Maximum stability was observed in presence of glutaraldehyde followed by isopropanol. Xylanase activity in presence of isopropanol has been reported using halophilic bacterium CL8 strain [23]. However, to the best of our knowledge, an activating effect of glutaraldehyde has not been observed for xylanases to date. Factors affecting the enzymes stability in organic solvents are changes in solvent-exposed surface areas and increase in the extent of secondary structure formation and truncated amino and carboxyl termini. Consequently, in surroundings with lower salt concentrations, the solubility of halophilic enzymes is often very poor which could limit their applicability [32]. However, this property makes enzyme stable in nonaqueous media [33, 34].

### 3.7. Storage Stability

Xylanases used in industrial applications are stored at different temperatures, that is, at room temperature, cooled, or frozen [34]. Enzyme retained 95% of activity at 4-5°C after storage for 1 month. Enzyme retained 85% of activity when stored at room temperature for 3 days. A 2-3 mL aliquot of xylanase was frozen for 3 weeks and residues of semisolid lyophilized enzyme retained nearly 60% of activity.

### 3.8. Application of Xylanase

All the lignocellulosic substrates, used for saccharification, were found to be susceptible for enzymatic hydrolysis as shown by a significant increase in the production of reducing sugars (Table 6). Reducing sugars were released from all agro residues following their treatment with the purified enzyme preparation. Corncob was saccharified more efficiently in comparison with wheat straw and rice straw after 24 hours, and the release of reducing sugars was increased with increase in the incubation period. The effect of xylanase treatment was more intensive on sugarcane bagasse pulp. Currently, industrial application of xylanases is in prebleaching of Kraft pulp in order to minimize the use of toxic chlorine-containing chemicals in the subsequent bleaching step [35, 36]. Since this xylanase could also saccharify natural lignocellulosic substrate, it seems to be a good candidate for use in the paper pulp industry to produce quality pulps. Optimization of xylanase by various statistical approaches is currently in progress.

## 4. Conclusion

The present work reports the characterization of haloalkali-moderately thermostable xylanase from newly isolated halophilic bacterium-OKH. It also addresses the property of xylanase such as stability in broad pH range, temperature, and NaCl concentration. Moreover, the ability of the strain halophilic bacterium-OKH to produce xylanase with agro residues supplements has been explored for economic xylanase production process. Application of purified xylanase in saccharification of agro residues was checked and efficient saccharification was found in sugarcane pulp after 24 hours of incubation. Thus, this strain could be good contender for different biotechnological applications under extreme conditions. Further, improvements in enzyme production using optimization parameters by statistical approach and use in biobleaching are in progress.

## Figures and Tables

**Table 1:** Morphological, physiological, and biochemical characteristics of halobacterium-OKH.

Character	Halobacterium-OKH
Colony characteristics	Translucent, slimy
Pigmentation	Cream pigmentation
Morphology	Gram-positive rods
Anaerobic growth	−
Motility	−
NaCl range	7–15%
NaCl optimum	12.5%
Temperature range	25–40°C
Temperature optimum	37°C
pH range	5.0–10.0
pH optimum	8.0
Catalase	+
Gelatinase test	−
Lipase test	−
Amylase test	+
Indole production	−
H_2_S production	−
Nitrate production	−
Sugar fermentation	
Glucose	+
Maltose	+
Sucrose	−
Mannitol	−
Xylose	+
Lactose	+
Antibiotic resistance	
Bacitracin	+
Metronidazole	+
Cefpodoxime	+
Teicoplanin	−
Streptomycin and pencillin G	+
Chloramphenicol	−
Tetracycline	+

**Table 2:** Effect of different sugars and agro residues as carbon sources for xylanase production.

Carbon sources	% Residual activity
Glucose	65
Maltose	45
Fructose	50
Mannose	52
Lactose	13
Arabinose	52
Galactose	43
Sucrose	12
Xylose	85
Oat spelt xylan	100
Birchwood xylan	94
Agro residues	
Rice Straw	29
Wheat Straw	72
Sugarcane bagasse	64
Corn cobs	87
Rice husks	42
Groundnut shells	25
Saw dust	21

**Table 3:** Purification of xylanase isolated from halobacterium-OKH.

	Total protein	Total activity	Specific activity	Fold purity	% yield
Crude enzyme	525.6	574.8	1.09	1	100
Ammonium sulphate precipitation	155.8	319.2	2.04	1.87	55.5
Ion exchange chromatography	42.3	185.9	4.39	4.02	32.3
Size exclusion chromatography	3.5	98.5	28.14	25.81	17.1

**Table 4:** Effect of metal ions and reducing agents on xylanase activity from halobacterium-OKH.

Metal ion/chemical	Relative activity (%)	
	2 mM	5 mM
Control	100	100
Ca^2+^	146	151
Ag^2+^	0	0
Hg^2+^	0	0
Co^2+^	65	63
Fe^3+^	0	0
Mg^2+^	119	125
Mn^2+^	138	142
Ni^2+^	121	107
Cu^2+^	0	0
Cd^2+^	0	0
Zn^2+^	0	0
Mercaptoethanol	126	117
EDTA	35	21

**Table 5:** Effect of organic solvents on xylanase activity. Solvents were used in 5% and 10%, respectively, and residual activity was recorded.

Solvents	Relative activity (%)	
	5%	10%
Methanol	11	0
Isopropanol	42	21
Acetone	15	0
Acetic anhydride	19	0
Glutaraldehyde	51	39

**Table 6:** Treatment of lignocellulosic substrate with purified xylanase.

Substrate	Reducing sugar (mg/mL)	
Lignocellulosic substrate	24 (h)	48 (h)
Corn cobs	2.45 ± 0.23	4.42 ± 0.36
Wheat straw	2.15 ± 0.76	3.87 ± 0.21
Rice Straw	1.89 ± 0.13	3.01 ± 0.91
Sugarcane bagasse	7.12 ± 0.63	11.69 ± 0.59

## References

[1] Dias A. A., Freitas G. S., Marques G. S. M. (2010). Enzymatic saccharification of biologically pre-treated wheat straw with white-rot fungi. *Bioresource Technology*.

[2] Ebringerová A., Heinze T. (2000). Xylan and xylan derivatives—biopolymers with valuable properties, 1. Naturally occurring xylans structures, isolation procedures and properties. *Macromolecular Rapid Communications*.

[3] Collins T., Gerday C., Feller G. (2005). Xylanases, xylanase families and extremophilic xylanases. *FEMS Microbiology Reviews*.

[4] Viikari L., Alapuranen M., Puranen T., Vehmaanperä J., Siika-Aho M. (2007). Thermostable enzymes in lignocellulose hydrolysis. *Advances in Biochemical Engineering/Biotechnology*.

[5] Sunna A., Antranikian G. (1997). Xylanolytic enzymes from fungi and bacteria. *Critical Reviews in Biotechnology*.

[6] Atomi H. (2005). Recent progress towards the application of hyperthermophiles and their enzymes. *Current Opinion in Chemical Biology*.

[7] Ventosa A., Nieto J. J., Oren A. (1998). Biology of moderately halophilic aerobic bacteria. *Microbiology and Molecular Biology Reviews*.

[8] Sánchez-Porro C., Martín S., Mellado E., Ventosa A. (2003). Diversity of moderately halophilic bacteria producing extracellular hydrolytic enzymes. *Journal of Applied Microbiology*.

[9] Adams M. W. W., Perler F. B., Kelly R. M. (1995). Extremozymes: expanding the limits of biocatalysis. *Bio/Technology*.

[10] Ventosa A., Nieto J. J. (1995). Biotechnological applications and potentialities of halophilic microorganisms. *World Journal of Microbiology and Biotechnology*.

[11] Mamo G., Hatti-Kaul R., Mattiasson B. (2006). A thermostable alkaline active endo-*β*-1-4-xylanase from Bacillus halodurans S7: purification and characterization. *Enzyme and Microbial Technology*.

[12] Giridhar P. V., Chandra T. S. (2010). Production of novel halo-alkali-thermo-stable xylanase by a newly isolated moderately halophilic and alkali-tolerant *Gracilibacillus* sp. *TSCPVG*. *Process Biochemistry*.

[13] De Paula Silveira F. Q., De Melo I. S., Filtho E. X. F. (1997). Carbohydrate-hydrol ysing enzyme activity production by soid state cultures of *Trichoderma harzianum* strains. *Revista de Microbiologia*.

[14] Buchanan R. E., Gibbons N. E., Cowan S. T. (1974). *Bergey’s Manual of Determinative Bacteriology*.

[15] Sambrook J., Russel D. W. (2001). *Molecular Cloning, A Laboratory Manual*.

[16] Miller G. L. (1959). Use of dinitrosalicylic acid reagent for determination of reducing sugar. *Analytical Chemistry*.

[17] Lowry O. H., Rosebrough N. J., Farr A. L., Randall R. J. (1951). Protein measurement with the Folin phenol reagent. *The Journal of Biological Chemistry*.

[18] Laemmli U. K. (1970). Cleavage of structural proteins during the assembly of the head of bacteriophage T4. *Nature*.

[19] Hung K. S., Liu S. M., Tzou W. S. (2011). Characterization of a novel GH10 thermostable, halophilic xylanase from the marine bacterium *Thermoanaerobacterium saccharolyticum* NTOU1. *Process Biochemistry*.

[20] Lineweaver H., Burk D. (1934). The determination of enzyme dissociation constants. *Journal of the American Chemical Society*.

[21] Goyal M., Kalra K. L., Sareen V. K., Soni G. (2008). Xylanase production with xylan rich lignocellulosic wastes by a local soil isolate of *Trichoderma viride*. *Brazilian Journal of Microbiology*.

[22] Prakash S., Veeranagouda Y., Kyoung L., Sreeramulu K. (2009). Xylanase production using inexpensive agricultural wastes and its partial characterization from a halophilic *Chromohalobacter* sp. TPSV 101. *World Journal of Microbiology and Biotechnology*.

[23] Wejse P. L., Ingvorsen K., Mortensen K. K. (2003). Purification and characterisation of two extremely halotolerant xylanases from a novel halophilic bacterium. *Extremophiles*.

[24] Menon G., Mody K., Keshri J., Jha B. (2010). Isolation, purification, and characterization of haloalkaline xylanase from a marine Bacillus pumilus strain, GESF-1. *Biotechnology and Bioprocess Engineering*.

[25] Amaya-Delgado L., Vega-Estrada J., Flores-Cotera L. B., Dendooven L., Hidalgo-Lara M. E., Montes-Horcasitas M. C. (2006). Induction of xylanases by sugar cane bagasse at different cell densities of *Cellulomonas flavigena*. *Applied Microbiology and Biotechnology*.

[26] Gupta S., Bhushan B., Hoondal G. S. (2000). Isolation, purification and characterization of xylanase from *Staphylococcus sp.* SG-13 and its application in biobleaching of kraft pulp. *Journal of Applied Microbiology*.

[27] Wejse P. L., Ingvorsen K., Mortensen K. K. (2003). Xylanase production by a novel halophilic bacterium increased 20-fold by response surface methodology. *Enzyme and Microbial Technology*.

[28] Manikandan K., Bhardwaj A., Gupta N. (2006). Crystal structures of native and xylosaccharide-bound alkali thermostable xylanase from an alkalophilic *Bacillus* sp. NG-27: structural insights into alkalophilicity and implications for adaptation to polyextreme conditions. *Protein Science*.

[29] Ratanakhanokchai K., Kyu K. L., Tanticharoen M. (1999). Purification and properties of a xylan-binding endoxylanase from alkaliphilic *Bacillus* sp. strain K-1. *Applied and Environmental Microbiology*.

[30] Bataillon M., Cardinali A. P. N., Castillon N., Duchiron F. (2000). Purification and characterization of a moderately thermostable xylanase from Bacillus sp. strain SPS-0. *Enzyme and Microbial Technology*.

[31] Sellek G. A., Chaudhuri J. B. (1999). Biocatalysis in organic media using enzymes from extremophiles. *Enzyme and Microbial Technology*.

[32] Madern D., Ebel C., Zaccai G. (2000). Halophilic adaptation of enzymes. *Extremophiles*.

[33] Marhuenda-Egea F. C., Bonete M. J. (2002). Extreme halophilic enzymes in organic solvents. *Current Opinion in Biotechnology*.

[34] Shah A. R., Madamwar D. (2005). Xylanase production under solid-state fermentation and its characterization by an isolated strain of *Aspergillus foetidus* in India. *World Journal of Microbiology and Biotechnology*.

[35] Bajpai P. (1999). Application of enzymes in the pulp and paper industry. *Biotechnology Progress*.

[36] Beg Q. K., Kapoor M., Mahajan L., Hoondal G. S. (2001). Microbial xylanases and their industrial applications: a review. *Applied Microbiology and Biotechnology*.

